# Implementing a balanced scorecard-driven performance dashboard for governance in a public oncology centre in Morocco

**DOI:** 10.4102/hsag.v31i0.3411

**Published:** 2026-05-20

**Authors:** Ismail Bejja, Adil Najdi

**Affiliations:** 1 Laboratory of Cancer and Chronic Disease Control, Faculty of Medicine and Pharmacy, Abdelmalek Essaâdi University, Tangier, Morocco

**Keywords:** balanced scorecard, performance management, oncology services, digital dashboard, performance indicators, Morocco

## Abstract

**Background:**

Cancer care in low- and middle-income countries (LMICs), including Morocco, is constrained by fragmented information systems, non-standardised indicators and limited integration of clinical, financial and administrative data. These weaknesses undermine governance capacity, impair care coordination and delay evidence-informed decision-making.

**Aim:**

To co-design and implement a balanced scorecard (BSC)-based digital performance governance model in a public university oncology centre in northern Morocco.

**Setting:**

The study was conducted at a public tertiary oncology centre integrated into Morocco’s National Cancer Control Programme.

**Methods:**

A qualitative participatory action research (PAR) design was used. Multidisciplinary workshops and semi-structured interviews supported the development of a BSC framework and the validation of 21 performance indicators across five perspectives: clinical results, financial sustainability, patient experience, internal processes and learning and growth. Patient-reported outcome measures (PROMs) and patient-reported experience measures (PREMs) were integrated into a digital dashboard. Descriptive statistics and thematic analysis were conducted within the PAR and BSC framework.

**Results:**

Operational indicators, including treatment completion and equipment utilisation, were high (≈90%), reflecting strong technical capacity. Diagnostic delays and late-stage presentation remained frequent. Patient-centred processes were weaker: satisfaction with care coordination and supportive services was suboptimal, and coverage of psychosocial and nutritional support was limited. Learning and growth indicators showed moderate staff training and restricted access to innovation.

**Conclusion:**

The BSC-based digital model improved transparency, coordination and strategic alignment, representing a replicable and contextually adaptable governance approach for oncology services in LMICs.

**Contribution:**

This study provides a replicable framework linking clinical performance, patient experience and organisational learning.

## Introduction

Cancer represents a major public health burden in Morocco and other low- and middle-income countries (LMICs), where cancer services continue to face fragmented information systems, non-standardised indicators and limited integration of clinical, managerial and financial data. These gaps weaken institutional governance and delay strategic decision-making (Kharraz, Ansari & Doukkali 2023). National assessments in Morocco have similarly highlighted persistent shortcomings in performance monitoring, care coordination and accountability in oncology centres. Comparable patterns are reported across African health systems, where late diagnosis, uneven service quality and limited resources continue to limit equitable cancer care delivery (Brand, Chao & Ilbawi 2020; McLeod et al. 2024).

The balanced scorecard (BSC) provides a structured framework for integrating clinical outcomes, internal processes, patient experience, financial performance and organisational learning.

Originally developed as a strategic performance management tool, the BSC has been widely adapted to public-sector and healthcare contexts, where it has demonstrated value in improving transparency, teamwork and strategic alignment (Inamdar & Kaplan 2002; Kaplan & Norton 1992, 2001; Zelman, Pink & Matthias 2003). Its multidimensional logic is particularly relevant for cancer systems in LMICs, where weaknesses in coordination, indicator fragmentation and data integration hinder comprehensive performance assessment and governance (Kharraz et al. 2023).

Digital dashboards offer additional opportunities to consolidate heterogeneous information streams and support more responsive and evidence-informed governance. Recent studies show that digital performance tools can improve decision-making, accountability and organisational learning when embedded within a coherent strategic and governance framework (Gotsadze et al. 2024; Katapally & Ibrahim 2023). However, in the absence of a unifying conceptual model, digital solutions often generate fragmented or non-actionable insights, limiting their contribution to strategic decision-making. Integrating BSC with a participatory dashboard development process can, therefore, strengthen decision-making, accountability and service coordination within public cancer centres, as suggested by recent African governance and performance management studies (Marutha & Ngulube 2020; Makhado 2023).

Together, these gaps highlight the need for an integrated and participatory performance governance framework capable of linking care processes, outcomes and organisational learning within cancer services.

### The purpose and objectives of the study

The purpose of this study was to co-develop and implement a BSC-based performance governance framework for a public oncology centre in Morocco, supported by a digital dashboard that integrates clinical, patient-reported, financial and organisational indicators. In line with this goal, the study sought to identify and prioritise context-relevant performance indicators through a participatory process involving clinical, administrative and managerial stakeholders; integrate clinical, financial and patient-reported data into an interactive digital dashboard and analyse performance patterns across the five BSC perspectives to highlight strengths, gaps and governance priorities within the cancer care pathway.

## Research methods and design

### Study design and setting

A qualitative participatory action research (PAR) design was adopted to strengthen performance governance within a real-world public oncology service. This design was selected because it facilitates organisational learning, stakeholder participation and iterative improvement (Makhado 2023; Marutha & Ngulube 2020).

The study followed the Plan–Act–Observe–Reflect framework (Kemmis & McTaggart 2005) and was implemented across two iterative PAR cycles, as documented in Online Appendix 1: Table 1–A1. Data were collected between July 2024 and June 2025 at the Tangier University Oncology Centre, a public tertiary hospital operating within Morocco’s National Cancer Control Plan.

### Stakeholder engagement and participants

A participatory process was used to co-develop and validate performance indicators. Four multidisciplinary workshops (15–20 participants each) and eight semi-structured interviews were conducted with clinicians, nurses, medical physicists, pharmacists and administrative staff. Participants were purposively selected to ensure representation of key functions throughout the cancer care pathway and governance processes, including clinical care delivery, supportive care, coordination and logistics and administration and finance. Participants were eligible if they held an operational role in the delivery or governance of cancer services and had direct participation in care processes and/or access to or routine use of data sources relevant to the indicators under discussion.

Interview participants were selected among individuals with decision-making responsibilities and/or ownership of routine datasets used for performance monitoring.

This approach aligns with evidence demonstrating that participatory performance-management processes improve teamwork, accountability and decision-making in African public hospitals (Makhado 2023; Tshitangano & Mafukata 2022).

### Participatory action research process

The study followed an iterative participatory process aligned with the planning–action–observation–reflection logic of PAR, implemented in two cycles. The process unfolded in five analytical phases: Context analysis, indicator generation, validation and strategy mapping, operationalisation and dashboard development. The correspondence between these phases and the PAR cycles is documented in Online Appendix 1: Table 1–A1.

#### Phase 1: Context analysis

A documentary review was conducted to cover the established organisational and strategic context, drawing on national strategic frameworks (Ministry of Health Morocco 2020), institutional activity reports, patient flow data, equipment inventories and international oncology benchmarks (World Health Organization [WHO]; International Atomic Energy Agency [IAEA]). Full details of the data sources and outputs are provided in Online Appendix 1: Table 1–A1.

#### Phase 2: Indicator generation and preliminary selection

Interactive workshops and semi-structured interviews were conducted to generate an initial list of 52 performance indicators. Workshops and interviews were structured using a seven-column participatory analysis grid (GAPOP) to ensure methodological continuity throughout data collection phases (Online Appendix 1: Table 1–A1). The selection criteria included relevance to local priorities, feasibility of measurement, alignment with international standards, where applicable, and managerial actionability. The indicator development process was informed by BSC principles adapted to public-sector health systems in LMICs (Kaplan & Norton 1992; Zerhouni, Alaoui & Mahmal 2022).

#### Phase 3: Indicator validation and strategy mapping

Indicators were refined through iterative consensus-building using a Nominal Group Technique (NGT)-based scoring process and were organised into the five perspectives of the BSC. Candidate indicators were evaluated against four predefined criteria: Relevance, feasibility, actionability and BSC alignment. This process resulted in 21 core indicators that were incorporated into a BSC strategy map illustrating hypothesised causal relationships linking resources, internal processes and outcomes, consistent with the BSC causal logic (Aidemark 2001; Kaplan & Norton 2001). The NGT scoring rules and indicator retention criteria are documented in Online Appendix 1: Table 1–A1.

An indicator – cervical cancer treatment completion – is supported by a formally published quality indicator framework that specifies the overall treatment time (OTT) ≤ 56 days and the completion of brachytherapy as a mandatory process quality indicator (Chargari et al. 2023) and is classified accordingly in Online Appendix 1: Table 2–A1.

Benchmark thresholds for non-clinical indicators were established through this participatory process. Where no universally standardised cut-off was identified in the published literature, thresholds represent consensus targets informed by domain literature rather than published standards, as documented in Online Appendix 1: Table 3–A1.

#### Phase 4: Data collection and indicator operationalisation

Multiple data sources were used to ensure triangulation:

Clinical data (*n* = 276): Diagnostic and treatment delays, treatment completion rates, treatment response, acute toxicity and stage at diagnosis:

- Administrative and financial data: Equipment utilisation, reimbursement rates and avoidable hospitalisation
- Patient-reported outcome measures (PROMs): Functional Assessment of Cancer Therapy-General (FACT-G) questionnaire (*n* = 200)
- Patient-reported experience measures (PREMs): Adapted electronic satisfaction (e-Satis) and oncologists’ satisfaction (Onco-Satis) questionnaires
- Acute toxicity was graded using the Radiation Therapy Oncology Group (RTOG) and the European Organisation for Research and Treatment of Cancer (EORTC) criteria (Cox, Stetz & Pajak 1995).

The clinical cohort included both female and male patients, depending on the tumour group. The breast and cervical cancer cases were only female, while the nasopharyngeal and lung cancer cases included patients of both sexes.

Clinical indicators were calculated at the service level and, where relevant, separately calculated for four groups of tumours (breast, cervix, nasopharynx and lung) to support clinically meaningful interpretation and benchmarking.

Patient-reported outcome measures and patient-reported experience measures were collected using adapted questionnaires informed by established and internationally used patient-experience frameworks. Items were contextually adapted through multidisciplinary workshops to reflect local care pathways, literacy levels and service organisation. These measures were used for organisational performance monitoring rather than for individual clinical assessment. Patient-reported outcome measures and PREMs were collected face to face at the oncology centre, consistent with recommendations for patient-centred performance evaluation in African health systems (Mothiba, Tladi & Mbombi 2021).

Because universally validated benchmark thresholds are not available for several oncology performance indicators, particularly in LMIC settings, institutional results were compared with reference ranges derived from international clinical guidelines, multicentre radiotherapy trials, and population-based cancer registries (Surveillance, Epidemiology, and End Results [SEER]; Global Cancer Observatory [GLOBOCAN]). These values are presented as contextual reference ranges rather than formal performance standards (Online Appendix 1: Table 2–A1).

#### Phase 5: Dashboard development

The validated indicators were integrated into a digital dashboard developed using Power BI Desktop (Microsoft Corporation 2024). Indicator measurement yielded 33 measured indicator instances, as selected clinical indicators were calculated separately for four tumour groups, while other indicators were measured once at the service level.

In the reflection phase, preliminary indicator outputs and dashboard visualisations were reviewed with stakeholders to confirm interpretability and governance relevance and to inform minor refinements prior to final reporting.

### Data analysis

A mixed-methods integrative approach was applied within the PAR process. Qualitative data from workshops and interviews were thematically analysed using an inductive approach, with double coding to improve analytical credibility, focusing on recurring themes related to performance challenges, indicator relevance, feasibility and governance use.

Quantitative data were analysed using descriptive statistics, including means or medians, proportions and 95% confidence intervals where applicable. Calculations were performed in Python (pandas, numpy and scipy) within a Jupyter Notebook environment. Power BI Desktop (Microsoft Corporation 2024) was used for dashboard prototyping and interactive exploration.

Qualitative insights and quantitative indicators were jointly interpreted within the BSC framework to examine how governance processes translated into observable organisational outcomes.

### Rigour and trustworthiness

Rigour was supported through methodological triangulation across documentary sources, multidisciplinary workshops, semi-structured interviews, routine clinical and administrative datasets and PROM and PREM. Trustworthiness was addressed through credibility (triangulation and member checking), dependability (a documented methodological audit trail), confirmability (transparent indicator development and a decision log) and transferability (a thick contextual description of the institutional and health-system setting), as detailed in Online Appendix 1: Table 1–A1 (Lincoln & Guba 1985).

### Relevance to sub-Saharan African health systems

Although this study was conducted in Morocco, the governance challenges it addresses – including fragmented information systems, non-standardised indicators and limited integration of clinical and administrative data – are widely reported in public oncology services in sub-Saharan Africa and other LMIC settings (Makhado 2023; Tshitangano & Mafukata 2022).

### Ethical considerations

Ethical clearance to conduct this study was obtained from the Ethics Committee of the Faculty of Medicine and Pharmacy of Abdelmalek Essaâdi University, Tangier (No. AC157JT/2025). All procedures involving human participants complied with the Declaration of Helsinki and the WHO Ethical Guidance for Health Systems and Implementation Research. Written informed consent was obtained from all participants. All datasets were anonymised prior to analysis.

## Results

### Overview of result derivation and indicator set

**FIGURE 1:**
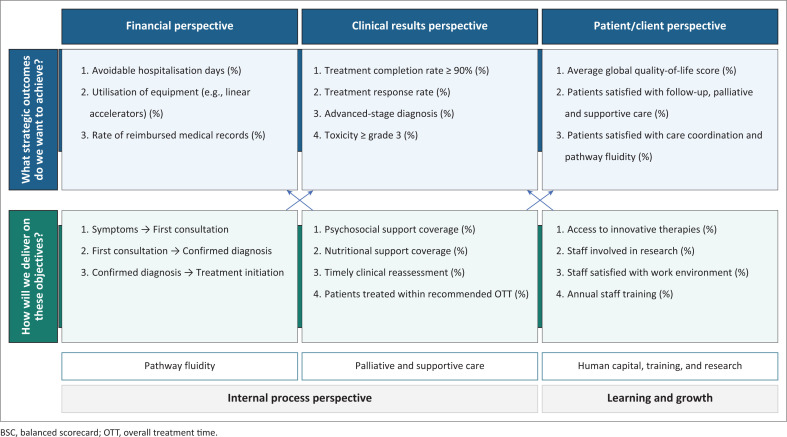
Balanced scorecard strategy map for the Study Oncology Centre (2024–2025).

The results are presented as: (1) the main outputs of the participatory process implementation (strategy map and validated indicator set) and (2) the performance findings measured across the five perspectives of the BSC. In line with the PAR process described in the Methods, the candidate indicators generated during the stakeholder workshops and interviews(*n* = 52) were prioritised and refined by iterative consensus against predefined criteria (relevance, feasibility, actionability and alignment with the perspectives of the BSC) to produce 21 core indicators represented in the strategy map (Figure 1).

For monitoring and benchmarking operational performance, the set of indicators was measured as 33 because selected clinical indicators were computed separately for four groups of tumours (breast, cervix, nasopharynx and lung). The operational definitions, benchmark typology and reference sources are reported in Online Appendix 1: Table 2–A1 (clinical indicators) and Online Appendix 1: Table 3–A1 (non-clinical indicators). Benchmark values were interpreted as contextual reference ranges and distributions rather than as fixed performance standards, acknowledging contextual differences in the mix of cases and resource availability. The use of standardised quality indicators to support performance benchmarking in LMIC oncology services has been increasingly recommended (McLeod et al. 2024).

Quantitative indicators were calculated from routine datasets and patient questionnaires and were jointly interpreted with qualitative insights within the BSC framework to identify governance priorities. The preliminary results were reviewed with stakeholders during the reflection phase to confirm interpretability and governance relevance prior to the final reporting.

### Balanced scorecard strategy map (implementation output)

A balanced scorecard (BSC) strategy map was developed through multidisciplinary workshops involving clinicians, managers, medical physicists, nurses and support care teams. The final framework comprises five perspectives – Clinical results, Financial Sustainability, Patient Experience, Internal Processes and Learning and Growth – linked through hypothesised causal pathways. This structure is consistent with the strategic logic of the BSC, which links organisational capabilities, internal processes and outcomes (Kaplan & Norton 1992, 2001).

The model illustrates how investments in human capital, digital readiness and innovation are expected to strengthen coordination and process performance, ultimately improving clinical results, patient experience and financial sustainability. This conceptual use of a BSC strategy map as a governance tool is consistent with prior applications in healthcare and LMIC public-sector settings (Aidemark 2001; Edward et al. 2011).

The full list of validated indicators, associated benchmarks and observed values is provided in Online Appendix 1: Table 2–A1 and Online Appendix 1: Table 3–A1.

Indicators validated through multidisciplinary consensus were organised into five perspectives and associated with exploratory causal pathways. The framework assumes that strengthening workforce competencies and digital capacity improves internal processes, which subsequently influence clinical outcomes, patient experience and financial performance. These causal relations remain hypothesised and are consistent with prior conceptual BSC applications in healthcare rather than empirically validated causal models (Zelman et al. 2003).

### Clinical results perspective

**FIGURE 2:**
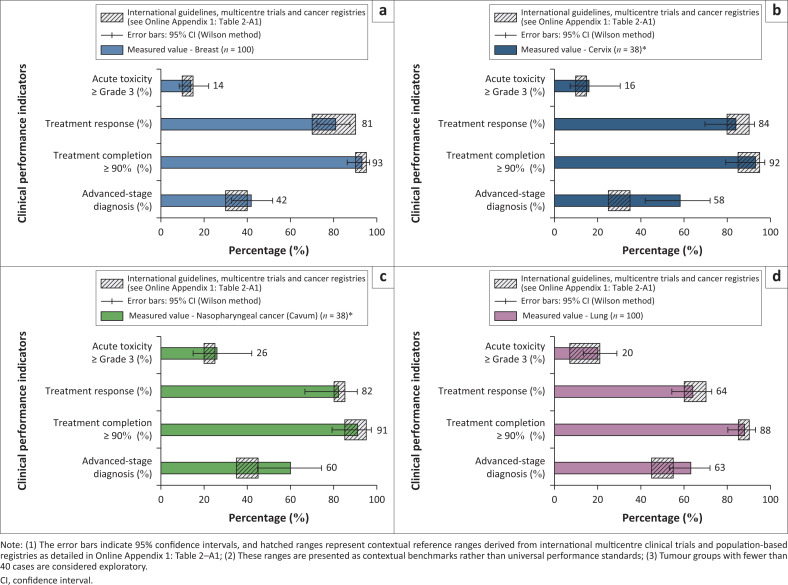
Clinical results indicators at the Study Oncology Centre (2024–2025). (a) Breast (*n* = 100) (Benchmarks vs study oncology centre); (b) Cervix (*n* = 38)* (Benchmarks vs study oncology centre); (c) Nasopharyngeal cancer (Cavum) (*n* = 38)* (Benchmarks vs study oncology centre); (d) Lung (*n* = 100), (Benchmarks vs study oncology centre).

The clinical outcome indicators for the four tumour groups are presented in Online Appendix 1: Table 2–A1. All acute toxicity indicators were graded using RTOG and EORTC criteria (Cox et al. 1995). Clinical performance varied between tumour groups (Figure 2). Treatment completion rates were high for all cancers (88% – 93%), meeting the operational performance targets derived from international radiotherapy practice recommendations (IAEA 2017). Treatment response rates were satisfactory for breast, cervical and nasopharyngeal cancers (81% – 84%) but notably lower for lung cancer (64%), a pattern frequently reported in LMIC oncology services (Brand et al. 2020). These comparisons are made against contextual reference ranges rather than strict performance thresholds, acknowledging contextual differences in case mix and resource availability.

Late-stage presentation was common at all tumour sites. The proportion of advanced-stage diagnosis exceeded international registry distributions for breast cancer (42% vs. ~30% – 40%), cervical cancer (58%), lung cancer (63% vs ~45% – 55%) and was consistent with predominantly advanced-stage presentation in nasopharyngeal cancer (cavum) in endemic North African settings (60%). For cervical cancer, the elevated proportion of advanced-stage diagnosis (58%) exceeds proportions reported in high-income registry data (SEER; GLOBOCAN), consistent with documented late-presentation patterns in LMIC settings and the epidemiological profile of cervical cancer in the African region (Bruni et al. 2023; Sung et al. 2021). Acute toxicity ≥ Grade 3 was highest for nasopharyngeal cancer (26%) and lung cancer (20%), patterns consistent with cisplatin-based concurrent chemoradiotherapy regimens (Sun et al. 2016). For breast cancer, the measured value (81%) represents the objective response rate (ORR) after neoadjuvant systemic therapy assessed by clinical and imaging criteria, not pathological complete response, and falls within the reported ORR range of ~70% – 90% (Cardoso et al. 2019).

Overall, clinical performance demonstrated strong treatment completion rates and satisfactory treatment responses across most tumour groups, but persistent challenges with early diagnosis and high toxicity rates in two tumour groups. These findings are consistent with performance patterns reported in comparable LMIC oncology services, where late-stage presentation and limited supportive care capacity contribute to the observed gaps (WHO 2020). Detailed indicator values, evidence categories, benchmark sources and contextual interpretations by tumour group are presented in Online Appendix 1: Table 2–A1.

### Financial perspective

**FIGURE 3:**
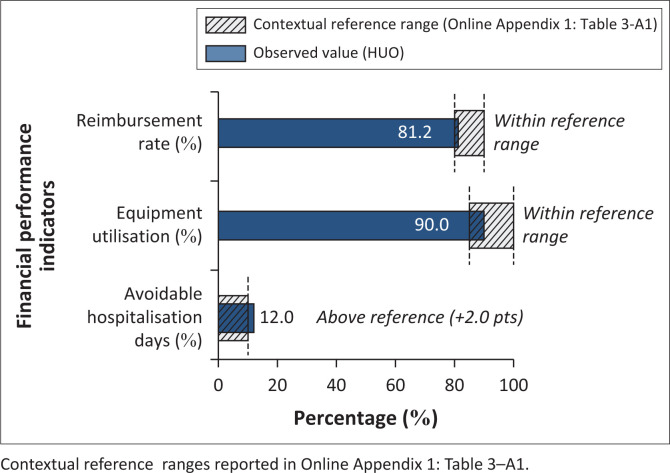
Financial performance indicators compared with contextual reference ranges (Study Oncology Centre, H2 2024–H1 2025).

The financial performance of the oncology centre showed a mixed yet informative profile across efficiency, resource utilisation and reimbursement processes (Figure 3). Equipment use reached 90%, within the contextual reference range (85% – 100%), indicating strong optimisation of radiotherapy capacity. Such high utilisation rates are commonly observed in public oncology centres facing high demand and limited capital resources (IAEA 2017; OECD 2021). These comparisons are made against internationally reported reference ranges rather than fixed efficiency standards, given the variability in financing mechanisms and case mix across health systems.

Reimbursement performance (81.2%) fell within the contextual reference range (80% – 90%), though remaining 8.8% points below the upper boundary, suggesting that while administrative processes are functional, there is room for improvement in claims management and documentation. In contrast, avoidable hospitalisation days (12%) exceeded the upper boundary of the contextual reference range (≤ 10%), signalling preventable inefficiencies in continuity of care and post-treatment follow-up. Similar inefficiencies have been reported in public oncology systems where outpatient coordination and early complication management are insufficiently developed (McLeod et al. 2024).

In general, the financial perspective highlights a dual reality: A strong use of capital-intensive assets alongside opportunities to reduce avoidable costs through improved clinical–administrative integration.

Horizontal bars show observed values for three indicators: Reimbursement rate, equipment utilisation rate and avoidable hospitalisation days. The hatched bars represent contextual reference ranges. Labels indicate whether the observed value falls within the reference range or, where applicable, the distance from the nearest boundary of the reference range.

### Patient-reported outcomes and experience

**FIGURE 4:**
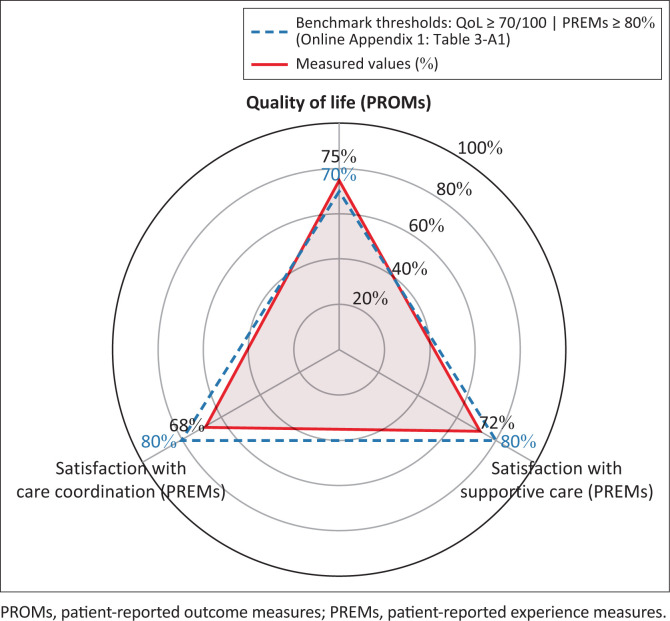
Patient-centred indicators (patient-centred outcome measures and patient-centred experience measures) at the Study Oncology Centre (2024–2025).

Patient-reported outcome measures and patient-reported experience measures are presented in Figure 4.

The mean global quality-of-life score reached 75/100, exceeding the operational benchmark (≥ 70/100) for comparable oncology settings (Cella et al. 1993; International Consortium for Health Outcomes Measurement [ICHOM] 2022). However, satisfaction with care coordination (68%) and supportive and follow-up care (72%) remained below the ≥ 80% benchmark threshold (OECD 2021; WHO 2020). This discrepancy reflects a recurrent pattern in LMIC oncology systems, where technical treatment delivery often outperforms patient-centred coordination and supportive care integration (Brand et al. 2020; WHO 2020). These patient-reported measures reflect perceived experience rather than objective service quality and may be influenced by expectations, communication practices and contextual factors specific to public oncology services.

The radar plot compares measured values for global quality of life (PROMs; FACT-G, Cella et al. 1993) and satisfaction with care coordination and follow-up and support (PREMs) against benchmark thresholds reported in Online Appendix 1: Table 3–A1 (Cella et al. 1993; ICHOM 2022; OECD 2021; WHO 2020). Benchmark thresholds applied were: Quality of life ≥ 70/100 and satisfaction indicators ≥ 80%. Δ values indicate deviations from these thresholds.

### Internal processes perspective

**FIGURE 5:**
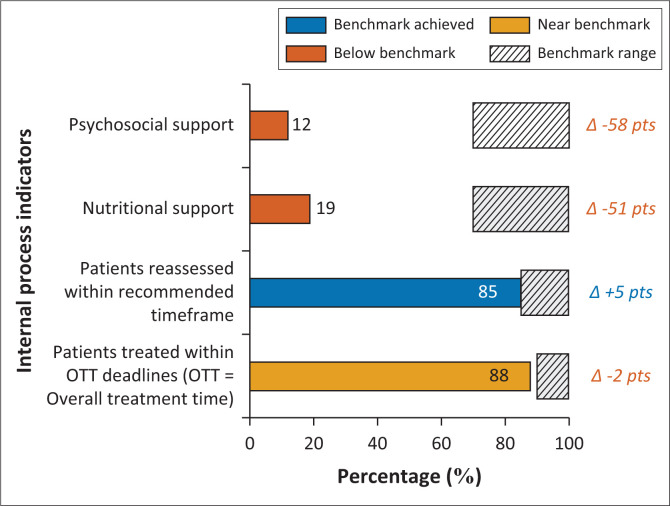
Internal process indicators compared to reference thresholds (Study Oncology Centre, H2 2024–H1 2025).

Internal process indicators revealed a heterogeneous profile in care-pathway coordination, timeliness and integration of supportive care (Figure 5). The timeliness indicators performed relatively well: 88% of patients-initiated radiotherapy within recommended OTT thresholds, and 85% received timely clinical reassessment, approaching the contextual benchmark targets (IAEA 2017; OECD 2021; WHO 2020).

In contrast, supportive care coverage was markedly limited. Only 19% of patients received nutritional support and 12% psychosocial support – far below participatory consensus benchmarks (WHO 2020). These gaps are consistent with structural constraints reported in many LMIC oncology centres, where multidisciplinary supportive care services remain under-resourced and weakly integrated (McLeod et al. 2024; WHO 2020).

Within a performance-governance perspective, this imbalance suggests that operational efficiency in treatment delivery has not been matched by equivalent investment in integrated supportive care pathways.

The benchmark thresholds are derived from IAEA (2017), OECD (2021) and WHO (2020); full details are reported in Online Appendix 1: Table 3–A1. Indicators were co-constructed through two PAR cycles (Online Appendix 1: Table 1–A1). Horizontal bars illustrate performance for four process indicators: (1) patients treated within OTT deadlines, (2) patients reassessed within recommended timeframes, (3) nutritional-support coverage and (4) psychosocial-support coverage. Grey-hatched intervals denote reference ranges, colour coding indicates the benchmark status and Δ values indicate deviations.

### Internal processes – Clinical delays

**FIGURE 6:**
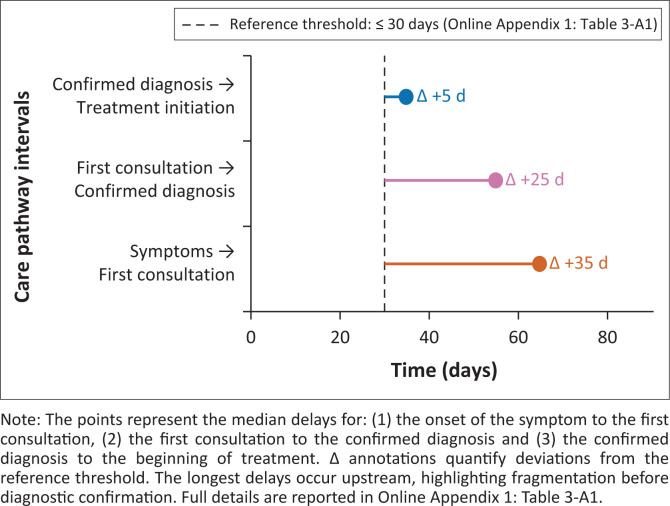
Clinical delays across the cancer care continuum (Study Oncology Centre, H2 2024–H1 2025).

The delays in the cancer care continuum remained substantial (Figure 6). The median interval from the symptom onset to the first consultation reached 65 days, more than double the commonly cited 30-day reference threshold (Online Appendix 1: Table 3–A1). The interval from first consultation to confirmed diagnosis was also prolonged (55 days), exceeding recommendations for timely diagnostic verification (OECD and European Commission 2026; WHO 2017).

These delays indicate persistent bottlenecks in early diagnostic pathways, particularly at the interface between primary care and specialist oncology services. Such patterns are well documented in LMIC health systems and contribute directly to late-stage presentation and poorer outcomes (Brand et al. 2020). From a governance perspective, these upstream delays reflect fragmented referral pathways and limited coordination mechanisms between care levels, underscoring the need for system-level performance monitoring beyond service-level indicators.

### Learning and growth perspective

**FIGURE 7:**
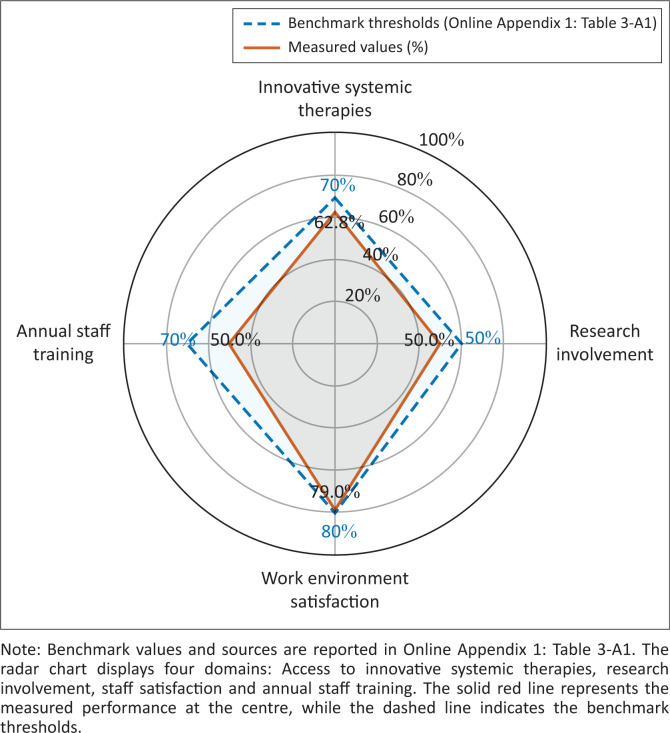
Learning and growth indicators at the Study Oncology Centre (2024–2025) compared with international benchmarks.

Indicators within the learning and growth perspective demonstrated uneven but generally moderate performance (Figure 7). Access to innovative systemic therapies (62.8%) meets the institutional benchmark (≥ 60%), while staff research involvement (50%) remained below the centre-specific target (≈60%), reflecting limited innovation capacity commonly reported in public oncology centres in LMICs (McLeod et al. 2024; Pramesh et al. 2022). Indicators are reported in Online Appendix 1: Table 3–A1.

Staff satisfaction approached the benchmark (79% vs. ≈80%), while annual training coverage remained modest (50% vs. 70%), highlighting persistent gaps in continuous professional development. Within the BSC logic, these learning and growth indicators represent enabling conditions rather than end results, as limitations in training, research engagement and access to innovation are likely to constrain future improvements in care coordination, patient experience and clinical results (McLeod et al. 2024; Pramesh et al. 2022).

The complete set of non-clinical indicators across the financial, patient-reported, internal process and learning perspectives, including benchmark typology and performance gaps, is reported in Online Appendix 1: Table 3–A1.

## Discussion

This study demonstrates that the BSC can be operationalised as a practical governance instrument within a public oncology centre in Morocco. Through a PAR process, the clinical, managerial and supportive care teams co-developed a set of 21 core indicators, operationalised them into 33 measured indicator instances and integrated them into a digital dashboard. This process strengthened the ownership of indicators and is consistent with African evidence showing that participatory approaches improve accountability, communication and teamwork in public hospitals (Makhado 2023; Tshitangano & Mafukata 2022). To our knowledge, this is the first study to design and implement a digitally supported BSC for oncology governance in a public hospital in North Africa, integrating clinical, financial, process and patient-reported metrics within a unified performance framework.

Beyond cancer, this study contributes to the broader health systems literature by demonstrating how a BSC can be operationalised as a governance and learning tool in complex public-sector settings characterised by fragmented data and resource constraints.

### Interpretation of the main findings

The findings indicate a mixed performance profile across the cancer pathway. The high treatment completion rates and the efficient use of radiation therapy reflect a strong technical capacity, which is a critical strength in LMIC oncology settings, where resource constraints often limit treatment delivery. However, persistent delays across the diagnostic continuum and the high proportion of late-stage presentations highlight structural weaknesses in referral systems, early detection and coordination – patterns widely documented across LMICs (Brand et al. 2020; WHO 2020).

A notable gap also emerged between technical treatment delivery and patient-centred supportive services. Patient-reported outcome measures and patient-reported experience measures showed that psychosocial, nutritional and navigation services remain insufficiently integrated into routine care. Similar discrepancies have been observed in African public hospitals, where supportive care is often underdeveloped despite adequate clinical capacity (Mothiba et al. 2021). These findings reinforce the need to analyse oncology performance beyond clinical outcomes and to consider the organisation of the care continuum.

Importantly, the BSC made these disparities visible by linking training, digital maturity and coordination processes with measurable outcomes. As suggested in previous research, the value of the BSC lies less in tracking metrics than in enabling organisational learning and shared interpretation of performance (Aidemark 2001; Zelman et al. 2003). The Learning and Growth perspective revealed training gaps, uneven access to innovation and limited digital readiness – factors that directly influence internal processes and patient experience.

These findings should be interpreted as early warning signals rather than performance failures, as limitations in learning and innovation capacity are likely to constrain future improvements across the care pathway.

This iterative process of co-construction and reflection, structured across two PAR cycles (Kemmis & McTaggart 2005), was essential to producing indicators that are both technically valid and organisationally meaningful.

These disparities are further detailed in Online Appendix 1: Table 1–A1, Table 2–A1 and Table 3–A1, which provide a structured overview of benchmark-based performance gaps across clinical and organisational dimensions.

### Implications for policy and practice

Three major governance implications emerge from this work.

Firstly, substantial diagnostic delays support calls for stronger early-detection governance, improved communication between primary and specialised care and systematic monitoring of diagnostic time-frames. Establishing referral standards and accountability mechanisms can reduce late-stage presentation, a persistent challenge in LMIC oncology.

Secondly, the limited availability of supportive and palliative care services underscores a structural deficit in multidisciplinary oncology. Because PROMs and PREMs strongly influence perceived quality, integrating psychosocial, nutritional and navigation services into daily practice should be a core governance priority.

Thirdly, the digital dashboard improved performance visibility and facilitated team discussions on coordination, resource use and bottlenecks. African studies increasingly show that digital performance tools can strengthen decision-making when anchored in participatory governance frameworks (Gotsadze et al. 2024; Katapally & Ibrahim 2023).

Together, these implications suggest a model of oncology governance that integrates technical capacity with supportive care, early detection and digital monitoring – an approach aligned with African health-system reform agendas.

### Strengths and limitations

A key strength of this study is the use of PAR, which allowed iterative refinement of indicators and promoted shared ownership among staff. The integration of PROMs and PREMs, which remain rare in LMIC oncology contexts, adds depth to performance assessment. The development of a theory-driven strategy map tailored to local realities further enhances applicability.

Limitations include the single-centre design, which limits generalisability; modest sample sizes for some tumour types and limited availability of long-term outcomes, such as survival, due to information system constraints. The study aimed to demonstrate the technical feasibility and governance utility of a BSC-driven digital dashboard rather than full-scale institutional deployment, which represents a further limitation of the current work. The clinical reference ranges reported in Online Appendix 1: Table 2–A1 were derived from multicentre trials and population-based registries, predominantly conducted in high-income settings. Their applicability to a public oncology centre in northern Morocco, operating under different resource, epidemiological and treatment-access conditions, is therefore limited. These values should be interpreted as contextual benchmarks rather than normative performance standards. In particular, elevated proportions of advanced-stage diagnosis reflect structural late-presentation patterns documented in LMIC oncology settings and should not be interpreted as indicators of suboptimal clinical care.

A further limitation concerns the benchmark thresholds used for non-clinical indicators (Online Appendix 1: Table 3–A1). Several thresholds represent participatory consensus targets co-constructed with local stakeholders rather than universally standardised cut-offs, reflecting both the PAR design and the limited availability of validated performance norms for oncology settings in Morocco and comparable LMIC contexts.

The participatory co-construction approach, reliance on routinely collected data and modular dashboard design were selected to enhance contextual relevance and facilitate adaptation in comparable resource-constrained health systems.

Nevertheless, the triangulation of clinical, administrative and patient-reported data improves credibility and enhances transferability to similar public oncology centres.

In general, the study provides evidence that a participatory and digitally supported BSC can strengthen governance, strategic alignment and performance monitoring in an LMIC oncology context. It achieves the initial objective of developing a practical data-driven performance framework aligned with national and international benchmarks. Future research should examine multicentre implementation, assess long-term outcomes and empirically test the causal pathways proposed in the strategy map to determine their impact on patient outcomes and organisational performance.

## Conclusion

This study demonstrates that a participatory digitally enabled BSC can be implemented as a practical governance tool within a public oncology centre in Morocco. The co-development and integration of clinical, organisational, financial and patient-reported indicators into a unified framework enabled structured performance evaluation, improved data visibility and the identification of critical gaps in care coordination and supportive services. In line with the study objectives, the findings highlight how a BSC-driven dashboard can support organisational learning, strengthen strategic alignment and inform governance priorities in resource-constrained oncology settings. Future research should explore multicentre implementation and examine the long-term effects of such governance models on care processes and patient outcomes.
